# Pilocarpine Induced Behavioral and Biochemical Alterations in Chronic Seizure-Like Condition in Adult Zebrafish

**DOI:** 10.3390/ijms21072492

**Published:** 2020-04-03

**Authors:** Yam Nath Paudel, Yatinesh Kumari, Syafiq Asnawi Zainal Abidin, Iekhsan Othman, Mohd. Farooq Shaikh

**Affiliations:** 1 Neuropharmacology Research Strength, Jeffrey Cheah School of Medicine and Health Sciences, Monash University Malaysia, Bandar Sunway, Selangor 47500, Malaysia; yam.paudel@monash.edu (Y.N.P.); yatinesh.kumari@monash.edu (Y.K.); iekhsan.othman@monash.edu (I.O.); 2 LC-MS/MS Laboratory, Jeffrey Cheah School of Medicine and Health Sciences, Monash University Malaysia, Bandar Sunway, Selangor 47500, Malaysia; syafiq.asnawi@monash.edu

**Keywords:** pilocarpine, zebrafish, seizure, cognition, inflammation, proteomics

## Abstract

Epilepsy is a devastating neurological condition exhibited by repeated spontaneous and unpredictable seizures afflicting around 70 million people globally. The basic pathophysiology of epileptic seizures is still elusive, reflecting an extensive need for further research. Developing a novel animal model is crucial in understanding disease mechanisms as well as in assessing the therapeutic target. Most of the pre-clinical epilepsy research has been focused on rodents. Nevertheless, zebrafish disease models are relevant to human disease pathophysiology hence are gaining increased attention nowadays. The current study for the very first time developed a pilocarpine-induced chronic seizure-like condition in adult zebrafish and investigated the modulation in several neuroinflammatory genes and neurotransmitters after pilocarpine exposures. Seizure score analysis suggests that compared to a single dose, repeated dose pilocarpine produces chronic seizure-like effects maintaining an average seizure score of above 2 each day for a minimum of 10 days. Compared to the single dose pilocarpine treated group, there was increased mRNA expression of HMGB1, TLR4, TNF-α, IL-1, BDNF, CREB-1, and NPY; whereas decreased expression of NF-κB was upon the repeated dose of pilocarpine administration. In addition, the epileptic group demonstrates modulation in neurotransmitters levels such as GABA, Glutamate, and Acetylcholine. Moreover, proteomic profiling of the zebrafish brain from the normal and epileptic groups from LCMS/MS quantification detected 77 and 13 proteins in the normal and epileptic group respectively. Summing up, the current investigation depicted that chemically induced seizures in zebrafish demonstrated behavioral and molecular alterations similar to classical rodent seizure models suggesting the usability of adult zebrafish as a robust model to investigate epileptic seizures.

## 1. Introduction

Epilepsy is a neurological disorder depicted by the spontaneous and unpredictable occurrence of seizures due to abnormal excessive and synchronous neuronal activity in the brain [1]. Much more is not known about the underlying mechanism of epileptogenesis but an ample amount of evidence suggests a pathogenic role of brain inflammation in epilepsy [2,3]. Neuroinflammatory mediators play a crucial role in the generation of seizure [4]. The high mobility group box 1 (HMGB1) is an initiator and amplifier of neuroinflammation and recently emerged as a novel frontier in epileptogenesis due to its plausible contribution to seizure propagation in animal models [5,6]. These evidences suggest that the HMGB1-toll like receptor -4 (TLR4) axis is implicated in epileptogenesis hence its targeting might have therapeutic utility against epileptogenesis [7].

More complex mammalian brains and genetic model organisms including zebrafish has been extensively studied that offers a significant benefit. Zebrafish (Danio rerio) has gained widespread popularity in behavioral neuroscience and psychopharmacology research [8]. In addition, the zebrafish model system offers large-scale screening and recapitulates the complexity of a whole-body organism, including the central nervous system (CNS) [9]. Interestingly, genetic compositions of zebrafish are comparable to humans with 70% of genetic similarity whereas 84% of genes known to human disease are widely expressed in zebrafish [10]. In addition, the zebrafish model is economically compared to rodents [11] which strengthens its usability to fit in the neuroscience research.

**Figure 1:**
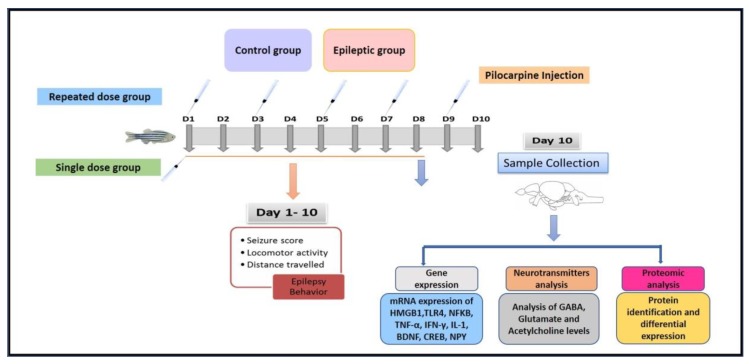
Experimental flowchart elucidating the working protocol.

Pilocarpine is the widely used pro-convulsant to induce chronic seizure-like state in rodents [12,13]. The mechanism of pilocarpine-induced status epilepticus (SE) depends on the activation of muscarinic M1 receptor and seizures are further retained by the activation of *N*-methyl-d-aspartate (NMDA) receptors [14]. Pilocarpine administration in rodents recapitulates the human temporal lobe epilepsy (TLE) features including limbic seizures, secondary generalized seizures, and SE that lasts for several hours [15]. Pentylenetetrazol (PTZ) [11] and kainic acid (KA) [16] induced seizure model already exists in adult zebrafish. In addition, pilocarpine has been earlier used as a pro-convulsant in zebrafish larvae [17,18]; however, no finding till date has reported pilocarpine induced seizure-like phenotype in adult zebrafish. In the current investigation, we attempted to develop pilocarpine-induced chronic seizure behavior and to investigate the modulation of several inflammatory genes (HMGB1, TLR4, NF-κB TNF-α, IL-1), neurotropic factor (BDNF), transcription factor (CREB-1), neuropeptides (NPY), neurotransmitters (GABA, Glutamate, and Acetylcholine) after single and repeated pilocarpine exposures. The modulation in expression of such inflammatory genes, neurotropic factor, transcription factor, neuropeptides, and neurotransmitters might provide insights about their contribution in the generation of an epileptic seizure. In addition, findings from the proteomic analysis provides information regarding the different identified proteins and its implication in the biological process (Figure 1). 

## 2. Results

### 2.1. Pilocarpine-Induced Seizure-Like Behavior

As per the standard protocol [19], seizure-like behavior as quantified by seizure scoring for pilocarpine-induced seizure has been developed via assessing the swimming pattern of epileptic zebrafish (Table 1).

**Figure 2:**
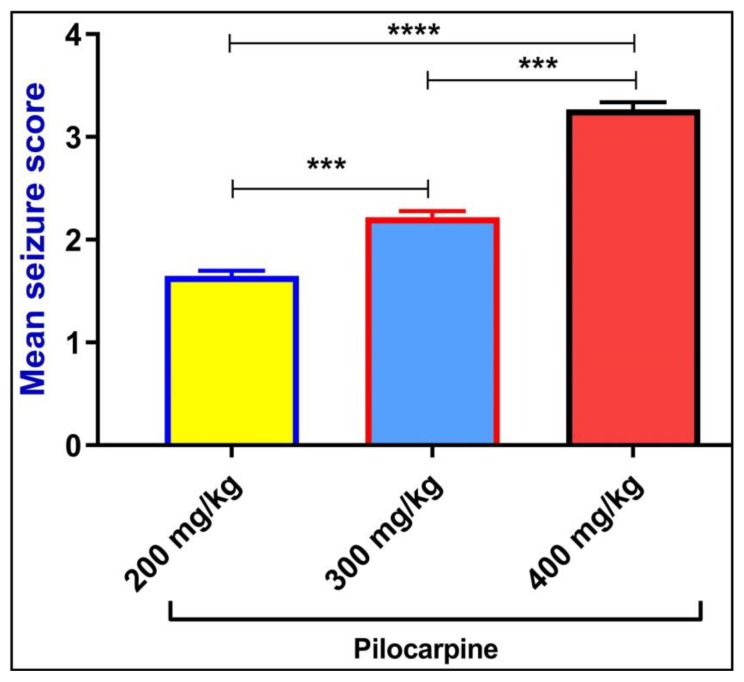
Dose standardization study of pilocarpine in adult zebrafish. All the values were expressed as mean ± SEM and each data point was the average of 8 fish in each group (*n* = 8). Statistical analysis was carried out using ANOVA. *P* < 0.05 was considered significant *p < 0.05, **p < 0.01, ***p < 0.001 and ****p < 0.0001.

Compared to the lower dose of pilocarpine (200 mg/kg) (****p < 0.0001) and medium dose of pilocarpine (300 mg/kg) (***p < 0.001), higher dose of pilocarpine (400 mg/kg) effectively produces seizure-like behavior as evident by significant increase in the seizure score. However, the seizure score produced by pilocarpine 300 mg/kg was significantly higher (***p < 0.001) as compared to the seizure score of pilocarpine 200 mg/kg (Figure 2). Based on the dose deciding studies, pilocarpine of dose 500 mg/kg has been discarded because of toxicity issues. Based on this observation, the epileptic dose of pilocarpine has been determined as 400 mg/kg.

### 2.2. Mean Seizure Score

**Figure 3:**
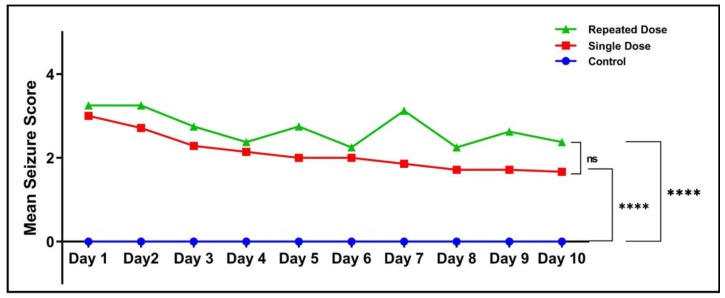
Comparison of mean seizure score. All the values were expressed as mean ± SEM and each data point where the average of 10 fish in each group (*n* = 10). Statistical analysis was carried out using two-way ANOVA, *p* < 0.05 was considered significant *p < 0.05, **p < 0.01, ***p < 0.001, ****p < 0.0001.

Overall, there was a significant difference (****p < 0.0001) in the mean seizure score between the control group and the single dose group. In addition, a significantly higher (****p < 0.0001) seizure score was observed in the repeated dose group when compared to the control group. However, no significant difference in the mean seizure score was observed between single and repeated dose pilocarpine group (Figure 3).

### 2.3. Total Distance Travelled, Time Spent in Upper and Lower Half of the Tank

**Figure 4:**
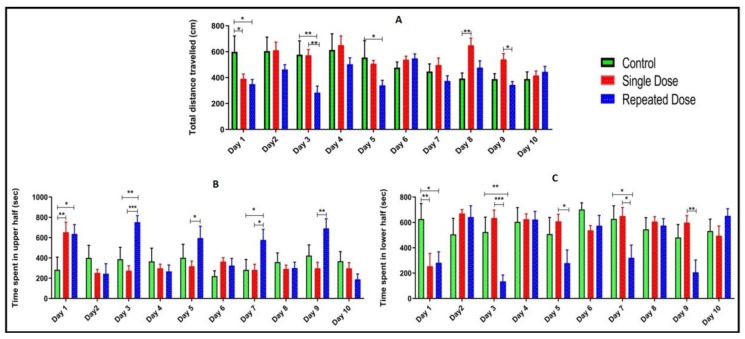
Total distance travelled, time spent in upper and lower half of the tank. All the values are expressed as mean ± SEM, and each data point shows the average of ten fish in each group (*n* = 10). Statistical analysis was carried out using two-way ANOVA, *p* < 0.05 was considered significant, *p < 0.05, **p < 0.01, ***p < 0.001. Total distance travelled in a tank (**A**), time spent in upper half (**B**), and time spent in lower half (**C**).

On the day of pilocarpine injection (days 1, 3, 5, 7, and 9), fish from repeated group traveled less distance in the tank compared to the fish from control and single dose group (Figure 4A). However, in day 1 there was a significant increase in the total distance travelled in the fish from control group compared to a single dose (*p < 0.05) and repeated dose group (*p < 0.05). On day 3, fish from normal group and single dose group demonstrated significant increase (**p < 0.01) in the total distance travel as compared to the fish from the repeated dose group. On day 5, fish from control group showed significant increase (*p < 0.05) in total distance travel as compared to the fish injected with repeated dose of pilocarpine. On day 8 and 9, epileptic fish from single dose group showed a significant increase in the total distance travelled as compared to the fish from control (**p < 0.01) and repeated dose (*p < 0.05) group respectively (Figure 4A).

On the day of pilocarpine injection (days 1, 3, 5, 7, and 9), epileptic fish injected with repeated dose of pilocarpine spent more time in the upper half of the tank compared to the fish from control and single dose pilocarpine injected group (Figure 4B). On day 1, single dose group spent more time in the upper half of the tank as compared to the control (**p < 0.01) repeated dose group (*p < 0.05). In addition, on the subsequent days (day 3, 5, 7, and 9) fish from repeated dose group spent more time in the upper half of the tank compared to the fish from control and single dose group (Figure 4B).

On the day of pilocarpine injection (days 1, 3, 5, 7, and 9), epileptic fish administered with repeated pilocarpine spent less time in the lower half of the tank as compared to the fish from control and single dose group (Figure 4C).

### 2.4. Comparison of Locomotor Pattern and Behavior

**Figure 5:**
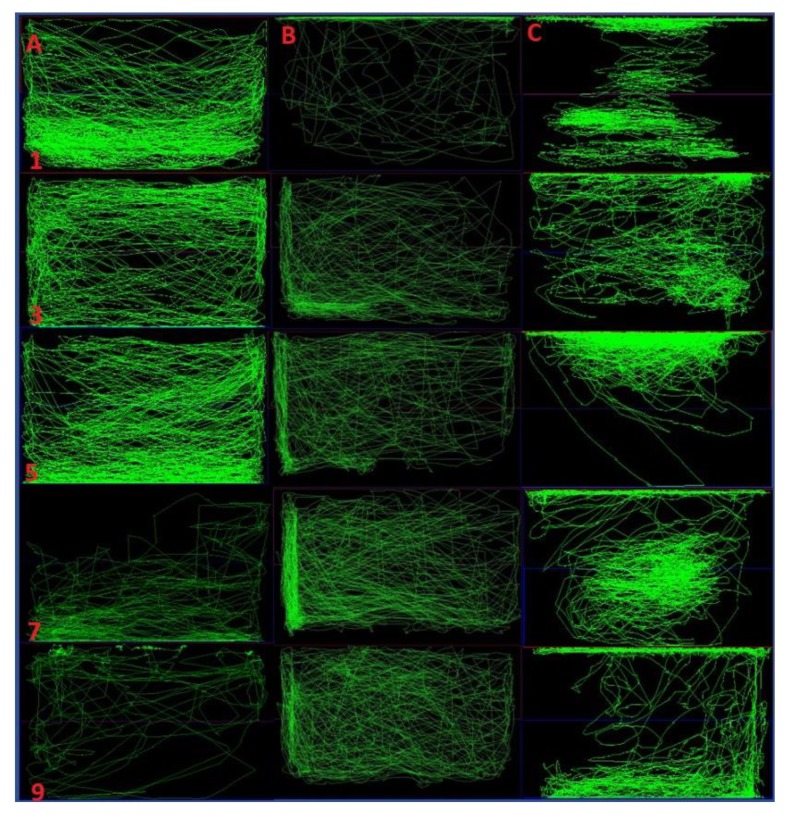
Comparison of locomotor pattern and behavior of fish. Single dose and repeated dose of pilocarpine treatment versus normal control. Representative swimming patterns (behavior recording) for the corresponding three experimental groups (**A**–**C**). The swimming pattern for the group (**A**) represents the 10% DMSO only vehicle control. The swimming pattern for the group (**B**) represents the single dose of Pilocarpine (400 mg/kg) whereas the swimming pattern for the group (**C**) represents the repeated dose of Pilocarpine (400 mg/kg) for 10 days. The number in the rows represents the swimming pattern of the specific day (1, 3, 5, 7, 9).

There was a similar swimming pattern (normal swimming all over the tank) in the control group throughout the experimental day (Figure 5, column A). On observation of locomotor pattern and behavior from the single dose group there was an abnormal tracking pattern due to seizure on day 1. In addition, there was a similar tracking pattern in the rest of the days (day 3, 5, 7, and 9) with hyperactivity on the certain portion of the tank (Figure 5, column B). However, in the fish treated with repeated dose pilocarpine, there was abnormal and disruptive swimming pattern on the day of pilocarpine administration (day 1, 3, 5, 7 and 9) (Figure 5 column C). This finding provide the notion that, as compared to the repeated dose of pilocarpine, single dose of pilocarpine does not strongly produce chronic effects for the duration of 10 days.

### 2.5. Modulation of Inflammatory Markers (HMGB1, TLR4, NF-κB, TNF-α, and IL-1), BDNF, CREB-1 and NPY upon Pilocarpine Exposure

#### 2.5.1. HMGB1

**Figure 6:**
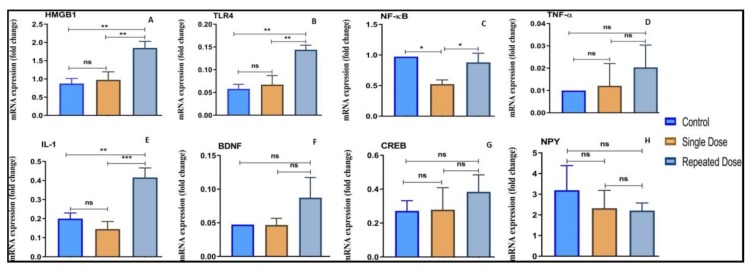
Modulation of inflammatory (HMGB1, TLR4, NF-κB, TNF-α, and IL-1) and other markers (BDNF, CREB-1, and NPY). Expression level of inflammatory (HMGB1, TLR4, NF-κB, TNF-α and IL-1) and other markers (BDNF, CREB-1, and NPY) in the brain as determined by real time-PCR. The genes included are (**A**) HMGB1, (**B**) TLR4, (**C**) NF-κB, (**D**) TNF-α, (**E**) IL-1, (**F**) BDNF, (**G**) CREB-1, and (**H**) NPY. All changes in the expression levels were compared to the repeated dose of pilocarpine (pilocarpine 400 mg/kg). Data are expressed as mean ± SEM, *n* = 6 and statistical analysis by one-way ANOVA followed by Sidak’s multiple comparison test, *p < 0.05, **p < 0.01, and ***p < 0.001.

There was a significant increase (**p < 0.01) in the mRNA expression level of HMGB1 in the repeated dose group as compared to the normal control group. In addition, there was a significant upregulation (**p < 0.01) in the mRNA expression of HMGB1 in the repeated dose group as compared to the single dose group. However, there were non-significant (ns) elevation in the single dose group when compared to the control group. The HMGB1 expression level for each group is graphically represented in Figure 6A.

#### 2.5.2. TLR4

Compared to the normal control and single dose group the repeated dose pilocarpine treated group exhibited significant upregulation (**p < 0.01) of TLR4 mRNA expression level (Figure 6B). However, the upregulation in the TLR4 mRNA expression level between normal control and single dose group were non-significant.

#### 2.5.3. NF-κB

Compared to the normal control group, the mRNA expression level of NF-κB surprisingly decreased in both the epileptic group injected with the single and repeated dose of pilocarpine (Figure 6 C). Compared to the normal group, there was a significant downregulation (*p < 0.05) in the mRNA expression level of NF-κB in the group treated with single dose pilocarpine. However, compared to the single dose group, there was significant elevation (*p < 0.05) in the NF-κB expression level in the repeated dose group as shown in Figure 6C.

#### 2.5.4. TNF-α

Compared to the normal control group, there was a non-significant elevation in the mRNA expression level of TNF-α in the single dose and the repeated dose of pilocarpine-treated group. Similarly, there was non-significant increment in the expression level of TNF-α in repeated dose group as compared to single dose group as represented in Figure 6D.

#### 2.5.5. IL-1

The mRNA expression level of IL-1 was significantly increased (**p < 0.01) in the group treated with repeated dose of pilocarpine when compared to the normal control group (Figure 6E). Similarly, there was a significant (**p < 0.01) increase in the expression level of IL-1 in the repeated dose group as compared to the single dose group. However, there was non-significant decrease in the expression level of IL-1 in the single dose group when compared to normal control group (Figure 6E).

#### 2.5.6. BDNF

The mRNA expression level of BDNF non-significantly increased in the group treated with pilocarpine when compared to the normal control group and single dose group (Figure 6F). However, there was no modulation in the expression level of BDNF in the single dose group as compared to the normal control group as shown in Figure 6F.

#### 2.5.7. CREB-1

Compared to the normal control group, the mRNA expression level of CREB-1 non-significantly increased in the single dose and repeated dose groups (Figure 6G). However, the changes were not statistically significant (Figure 6G). In addition, there was increment in the mRNA expression level of CREB-1 in the repeated dose group compared to the single dose group, however the changes were not significant (Figure 6G).

#### 2.5.8. NPY

Compared to the normal control, there was non-significant decrease in the mRNA expression level of NPY in the single and repeated dose of pilocarpine-treated group respectively (Figure 6H). Moreover, compared to the single dose group, there was non-significant decline in the expression of NPY in the group injected with repeated dose of pilocarpine (Figure 6H).

### 2.6. Modulation of Neurotransmitters Levels in Zebrafish Brain

**Figure 7:**
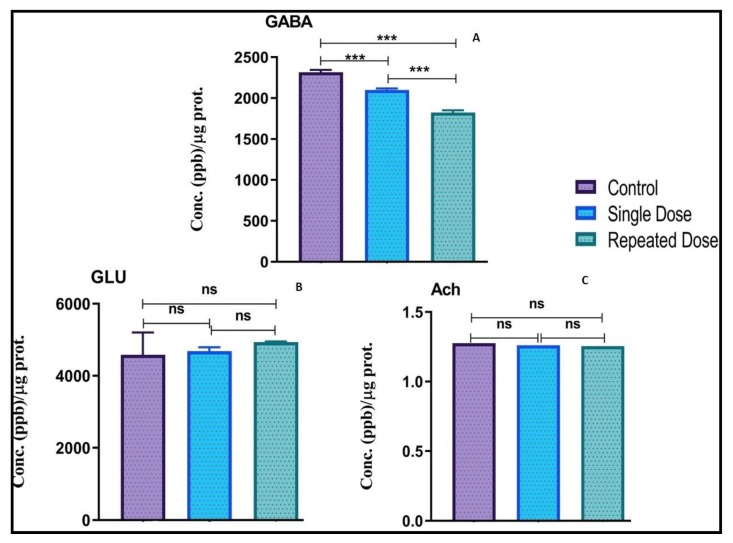
Modulation of Neurotransmitters levels in the normal and epileptic group. Neurotransmitters analysis in zebrafish brain after 10 days of pilocarpine treatment. GABA (**A**), Glutamate (GLU) (**B**), and Acetylcholine (Ach) (**C**) levels were estimated in the zebrafish brain using LC-MS/MS. Data are represented as mean ± SEM, *n* = 6 and statistically analyzed by one-way ANOVA followed by Sidak’s multiple comparison test *p < 0.05, **p < 0.01, and ***p < 0.001.

Neurotransmitter analysis by LC/MS-MS demonstrated significant downregulation in the level of GABA in the groups treated with single (***p < 0.001) and repeated dose of pilocarpine (***p < 0.001) when compared to the normal control group (Figure 7A). In addition, compared to the single dose group, repeated dose group demonstrated significant (***p < 0.001) downregulation in the level of GABA.

Glutamate level was elevated in single and repeated dose group as compared to normal control group; however, the elevation was non-significant (Figure 7B). As well as the increment in the level of Glutamate between single and repeated dose group were non-significant.

There was non-significant decrease in the level of Acetylcholine in the single and repeated dose group when compared to normal control group (Figure 7C). In addition, there was a downregulation in the level of Acetylcholine in the repeated dose group when compared to the single dose group; however, the changes were non-significant (Figure 7C).

### 2.7. Proteomic Analysis

**Figure 8:**
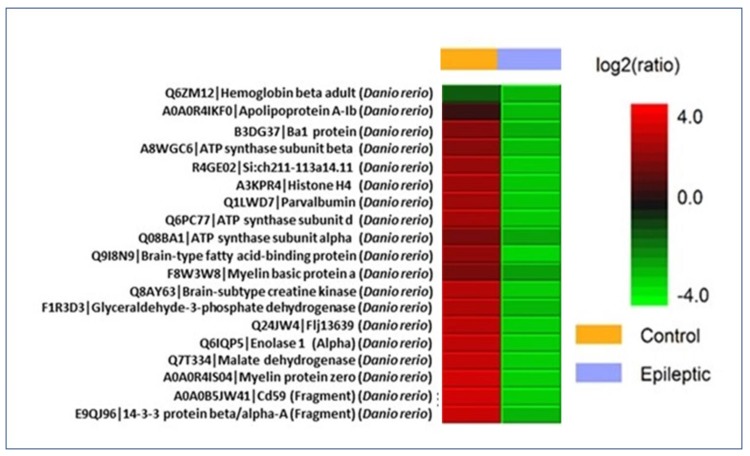
Differential expression (LFQ) of normal vs. epileptic zebrafish brain protein extract denoted by heat map. Label free quantification heat map for proteins identified from zebrafish brain (control vs. epileptic). Proteins with high ratio and low ratio are labelled red and green respectively. Samples are taken in triplicate where yellow line denotes epileptic group and light blue line denotes normal control group.

**Figure 9:**
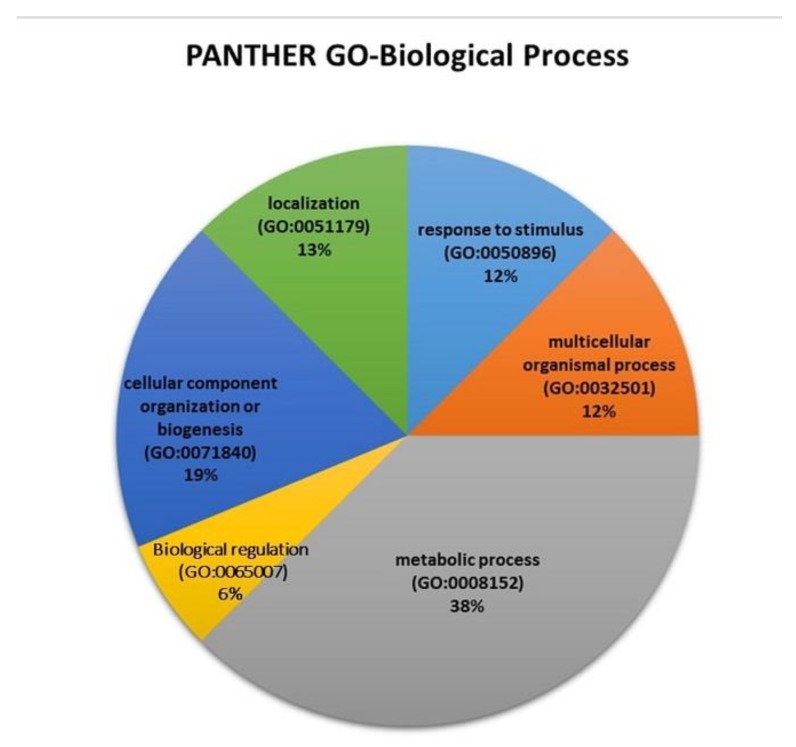
Biological process for the differentially expressed proteins. Biological processes for the differentially expressed proteins identified from the label free quantification approach. The pie chart was generated using PANTHER GO-classification system software.

The majority of protein identified in an epileptic zebrafish (13 proteins) are ependymin, keratin 91, keratin type 2 cytoskeletal, keratin 8, type 2 cytokeratin, keratin 5, LOC794362 protein, neurofilament medium polypeptide a, tubulin α and β chain, gamma1α-synuclein, and synuclein gamma β (Table 2). Whereas 77 proteins were identified by LCMS/MS in protein extract from normal zebrafish (Table 3). The −10lg*P* value signifies that higher the score, more confident is the detection of the protein and the −10lg*P* value for the protein identified in both the group is above 30. The value for peptides denotes that, these number of peptides belongs to the particular identified protein as mentioned in protein description. The value of unique signifies its resemblance with the identified protein. Coverage (%) means out of the identified peptides, only particular % belongs to the identified protein. Differential expression of protein-based on label free quantification suggests that the epileptic group has a low ratio of protein as compared to the normal group as evidenced in the heat map for protein (Figure 8). The proteins identified in the brain of zebrafish from both groups (epileptic and normal control) were observed to be directly allied with various metabolic process (38%), multicellular organismal process (12%), response to stimulus (12%), localization (13%), biological regulation (6%), and cellular component organization or biogenesis (19%) (Figure 9).

## 3. Discussion

In the current study we developed a chronic seizure-like condition using pilocarpine in adult zebrafish. Moreover, behavioral, proteomics, and molecular approaches have been undertaken to differentiate the changes in normal and epileptic zebrafish. Seizure score analysis after pilocarpine administration suggests that compared to the single dose, repeated dose of pilocarpine produces chronic seizure-like stage in an adult zebrafish for at least 10 days.

Majority of the epilepsy research till date has been performed in rodents as well as from human tissue obtained during surgical resection for intractable epilepsy [20]. However, zebrafish has emerged as a robust animal model for several neurological diseases including epilepsy [11,16,21]. Several findings are emerging that promote, suggest, and uplift the utilization of this underutilized laboratory species in neuroscience and neuropharmacology research [8,22]. Moreover, zebrafish models have contributed to a better understanding of the role of several genes that has been implicated in the disease [23]. Current investigation defines the epileptic dose of pilocarpine (400 mg/kg; i.p) that demonstrates the chronic seizure-like stage in adult zebrafish. Moreover, the seizure behavior for pilocarpine-induced seizure has been developed based on the comprehensive catalogue of zebrafish behavior [19]. On comparison of mean seizure score repeated dose of pilocarpine produces an average mean seizure score of 2 continuously till 10 days. Locomotor pattern demonstrated abnormal and disruptive swimming pattern on the day of pilocarpine administration.

Despite the dosing pattern of pilocarpine (single and repeated), the total distance travelled by epileptic fish is lower as compared to the control fish. Moreover, epileptic group (repeated dose) spent more time in the upper half of the tank on the day of pilocarpine administration compared to the next day of pilocarpine administration. Compared to the fish from control group, epileptic fish treated with repeated dose of pilocarpine spent less time in the lower half of the tank on the day of pilocarpine administration in comparison to the next day of pilocarpine administration. These findings from the behavioral study reflects the abruption of behavior upon pilocarpine administration. This observation was different from the PTZ-induced seizure behavior where PTZ-induced fish spent less time in upper half and more time in lower half of the tank after PTZ administration [21]. Moreover, this can be further speculated that different pro-convulsant might produce different behavioral changes compared to each other.

Neuroinflammation and modulation of pro-inflammatory cytokines lead to varying degrees of long-term alterations in the brain [24]. Experimental and clinical studies demonstrated that various mediators of inflammation are present in the brain, CSF, and blood in epileptic conditions [25]. The complex pathology of epilepsy still remained to be fully understood; however, accumulating evidence strongly supports the contribution of neuroinflammation in the pathophysiology of epilepsy [2,3,26]. To precisely understand the modulation of inflammatory mediators after pilocarpine exposure, the current investigation assessed the expression levels of several inflammatory markers (HMGB1, TLR4, NF-κB, TNF-α and IL-1) via gene expression studies.

HMGB1 has been implicated in the seizure generation via activation of its principal receptor mainly TLR4 [27]; however, the precise mechanism still remains less understood. Earlier finding reported increased mRNA expression of HMGB1 in a pilocarpine-induced epilepsy model in mouse [28]. Our finding reporting increased mRNA expression of HMGB1 in an epileptic group (repeated dose pilocarpine) is in similar line with earlier studies supporting the notion that HMGB1 might play a crucial role in the pathogenesis of epilepsy. The contribution of HMGB1 in seizure generation is mainly mediated by RAGE and TLR4. NF-κB is a crucial nuclear transcription factor important for innate and adaptive immunity. Several earlier findings have revealed an activation of TLR4/NF-κB signaling pathway in epilepsy which is evident by an increased level of TLR4 and NF-κB in epileptic animals as well as reflecting TLR4 and NF-κB inhibition as an therapeutic strategy for minimizing epileptic seizure [29,30,31]. Current study observed an elevated and downregulated mRNA expression of TLR4 (repeated dose pilocarpine) and NF-κB respectively in the epileptic group as compared to the normal control group. Our findings are in corroboration with earlier findings from rodents and clinical experimentation where TLR4 level has been reported to be upregulated in epileptic conditions [15,24,29]. Hence, current study speculates an activation of HMGB1/TLR4 signaling axis and absence of TLR4/NF-κB signaling pathway in pilocarpine-induced chronic seizure-like condition in adult zebrafish.

The effect of TNF-α on seizures depends mainly on its endogenous brain levels and the receptor subtypes predominantly stimulated by this cytokine [25]. Earlier findings reported upregulated level of TNF-α in epilepsy either in rodents [32] or in adult zebrafish [23]. Confirming the earlier finding, our study also reported increased level of TNF-α in an epileptic group injected with reported dose of pilocarpine. There is a conflicting finding about the role of IL-1 in epilepsy. Brain tissue from epilepsy patients and from experimental animal models reported increased IL-1 expression after seizures whereas exogenously applied IL-1 has pro-convulsive properties [33]. The mRNA expression level of IL-1 in the current study was reported to be increased in epileptic group treated with repeated dose of pilocarpine which was in similar line with earlier studies reporting upregulated IL-1 level in limbic status epilepticus [34]. Of importance, the increase in expression of several inflammatory markers in pilocarpine-induced chronic seizure in zebrafish reflects that inflammatory pathways play a crucial role in the incidence of epilepsy.

Seizure activity increases the expression of BDNF mRNA and protein reflecting that BDNF might contribute to epileptogenesis. This is evident by the findings reporting seizure induced increases in BDNF mRNA levels that peaks at 6 h after the seizure onset and return to control levels ~12 h after seizures termination [35]. Non-significant increase in BDNF mRNA level observed in epileptic group compared to normal group in our study also strengthens the possibility of BDNF demonstrating its role in seizure generation. CREB-1 exhibited a role in several biological process including the suppression of epilepsy [36] as well as long-term potentiation of memory [37]. The increased mRNA expression of CREB-1 in epileptic group compared to normal group reflects the plausible role of CREB-1 in epilepsy as well as suggests that memory is impaired in epileptic group. NPY is an endogenous peptide with powerful anti-convulsant properties [38]. Elevated level of NPY expression in brain regions is crucial for learning and memory together with its neuromodulatory and neurotrophic effects implicating a regulatory role for NPY in memory processes [39]. Current investigation observed downregulation in mRNA expression level of NPY in epileptic group compared to normal control group implicating the possibilities of learning and memory abnormalities in epileptic group.

GABA plays a crucial role in learning and memory [40] and is the primary neuroinhibitory in the central nervous system. GABA_A_ receptors signaling possess several context-specific activity that can prevent or promote epileptogenesis and seizure generation [41]. Reflecting the disruption in GABAergic system, current study demonstrated decreased level of GABA in the epileptic group when compared to normal control that supports the earlier similar findings [42]. Glutamate is an excitatory amino acid that has been implicated in the pathogenesis of epilepsy [43,44]. The release of glutamate might result in an increased intracellular calcium ultimately leading to cell death [45] as well as glutamate toxicity alters learning and memory [46]. Upregulated level of Glutamate in epileptic group observed in the current study which is in agreement with earlier study [21] speculates an evidence of cell death as well as altered learning and memory in the epileptic group. Acetylcholine plays a crucial role in regulating Glutamate release and maintaining memory formation [47]. Decreased level of Acetylcholine in both the epileptic groups compared to normal control group speculates an impairment of memory in a pilocarpine treated epileptic group.

Proteomic analysis provides insights about the alterations in the molecular pattern and helps in the identification of biomarkers associated with epileptogenesis [48]. Very few proteins identified in epileptic zebrafish brain extract as compared to the normal control group signifies that several proteins have been downregulated as well as biological process has been disrupted during the diseases condition. This is even supported by the differential expression of proteins based on label-free quantification (Table 4). Moreover, the identified protein in the epileptic group such as tubulin beta chain, tubulin alpha chain, neurofilament, α-synuclein has been implicated in epilepsy [49,50]. Proteomic analysis from Uniprot and (protein analysis through evolutionary relationships) PANTHER implicates that the identified proteome data set for zebrafish were majorly associated with metabolic processes. The obtained differential proteome and the direct association of the various proteins in normal and diseased (epileptic) zebrafish might increase our understanding about the expression of several proteins in an adult zebrafish.

Although the detrimental effects of this muscarinic agonist (pilocarpine) have been widely studied in rodents, the use of adult zebrafish to study epileptic seizures and is still in its early stages. This study posits that zebrafish has a huge potential to be modelled as an experimental animal model that can recapitulate the human epileptic behavior. Pilocarpine administration prompts seizures in adult zebrafish reflecting that adult zebrafish can be represented as a new model to investigate the mechanism of seizure generation. Current study strengthens this possibility by developing a chronic epilepsy-like condition in adult zebrafish upon pilocarpine exposure. Pilocarpine-induced epileptic zebrafish demonstrated the behavioral alterations, modulation of inflammatory markers and neurotransmitters level as seen in rodents model reflecting its usability in further research.

In a limiting part, the implication of the current study would have been better if pilocarpine-induced seizures and neuronal death have been justified with electrophysiological recordings and histopathological findings respectively.

Nevertheless, current study via modeling chronic seizure-like condition in an adult zebrafish using pilocarpine pave the way for further research. However, extensive further investigations are warranted to develop zebrafish as a robust model system to continue its use in understanding the mechanism of seizure generation, assessing new anti-consultants as well as evaluating the therapeutic potential of novel anti-epileptic therapy.

## 4. Material and Methods

### 4.1. Experimental Equipment and Chemicals

All analytical grade reagents were used unless specified otherwise. Water was purified and filtered by a specific LC-MS filter using a Milli-Q system from Millipore (Bedford, MA, USA). Pilocarpine was purchased from Sigma Aldrich (St. Louis, MO, USA). Formic acid (FA) was purchased from Friedemann Schmidt Chemicals, Parkwood 6147, Western Australia. Fish tank—10 L capacity (PETCO-Pet keeper, Malaysia). Sony Handycam (AVCHD 5x) recorder (Minato City, Tokyo, Japan), Sony Camcorder stand, Smart 3.0.05 tracking software (Pan Lab, Harvard apparatus), Hamilton syringe 700–702 series 25 µL, BD disposable needle (30G), Agilent Infinity 1290 UHPLC, coupled with Agilent 6410 Triple Quad LC/MS (Santa Clara, CA, USA), Applied Biosystems StepOnePlus™ Real-Time PCR Systems (Thermo Fisher Scientific, USA).

### 4.2. Zebrafish (Danio rerio) Care and Maintenance

Adult zebrafish (*Danio rerio*) of heterozygous wild-type-AB stock (standard short-fin phenotype) were obtained from IMCB, Institute of Molecular and Cell Biology, 61 Bioplis Drive Proteos, Singapore 138673. Both male and female fish were used in the ratio of 1:1 in all the experiments. All fish were kept in Monash University Malaysia fish facility at 28 °C, with a 10/14 h dark/light cycle (white incident light off at 10 pm, white incident light on at 8 am) under standard aquarium conditions. The care was taken to maintain system water pH between 6.8 and 7.1 by using electronic pH pen (Classic PH Pen Tester, Yi Hu Fish Farm Trading, Singapore 698950) and intensity of light was maintained at 250 lux to get the uniform light all over the housing area. The constant source of nourishment was ensured by feeding the fish twice a day. Nutrition for fish was maintained by Tropical TetraMin^®®^ Flakes and live brine shrimps artemia from Bio-Marine (Aquafauna, Inc. United States) three times a day with ad libitum feeding. Circulating water system with standard zebrafish tank, which is equipped with constant aeration having (36 cm × 26 cm × 22 cm) tank dimensions [51]. All the experiments were approved by Monash University Malaysia, animal ethics committee (MUM/2018/05).

### 4.3. Pilocarpine-Induced Seizure Behavior

The pro-convulsion dose of pilocarpine in an adult zebrafish is unknown hence, the initial work was to standardize the dose of pilocarpine in adult zebrafish. The dose of pilocarpine was standardized from the dose-deciding study where 200 mg/kg, 300 mg/kg, 400 mg/kg, and 500 mg/kg of pilocarpine was used (*n* = 8). Pilocarpine solution was prepared in the distilled water. Based on the outcomes of the dose-deciding study, higher dose of pilocarpine (500 mg/kg) has been discarded due to toxicity issues.

#### Experimental Design

After dose standardization, we designed a protocol to develop a pilocarpine-induced chronic seizure-like condition. The objective was to evaluate whether a single dose or repeated dose of pilocarpine produces chronic seizure-like condition in adult zebrafish for a minimum of 10 days. Normal control group (*n* = 12) only received distilled water. Single dose group (*n* = 12) received a single injection of pilocarpine (400 mg/kg) on day 1, whereas repeated dose group (*n* = 18) received repeated pilocarpine injection (400 mg/kg) on day 1, 3, 5, 7, and 9 as shown in experimental protocol (Figure 1). All the groups were used to record seizure behavior recording for 15 min each day post injection for 10 days as per protocol.

### 4.4. Epilepsy Behavior and Seizure Score Recording

Epileptic group was exposed to pilocarpine (400 mg/kg, i.p) demonstrated different seizure profiles, intensities, and latency to reach the scores. Seizure scores can be quantified manually (using seizure scale) or applying automated video-tracking tools (by assessing the velocity and distance travelled). Seizure score, seizure onset, total distance travelled, time spent in upper and the lower half of the tank were also noted for 10 days. Side-view recording of observation tanks was used for the recording of seizure-like responses in adult zebrafish.

### 4.5. Gene Expression Studies

#### 4.5.1. Brain Harvesting

Zebrafish brains were harvested at the end (day 10) of behavior study to determine the molecular changes in the brain. The brains from each group were divided into two halves and each brain was then transferred into trizole to check gene expression levels and another half into methanol for LC-MS/MS studies. The whole process of brain harvesting was done on ice and the brain was immediately frozen at −65 °C in dry ice. The soft skull of the fish was removed first and then the whole brain was extracted with the help of forceps and placed in respective solvent. All the brains were stored at −80 °C until further use. Gene expression studies were carried out to investigate the modulation of several genes (HMGB1, TLR4, NF-κB TNF-α, IL-1, BDNF, CREB-1, and NPY) in normal control and epileptic group injected with single and repeated dose of pilocarpine. All the brain samples were collected in ice-cold 200 µL TRIzol^®^ reagent (Invitrogen, Carlsbad, CA, USA) and immediately stored at −80 °C until further usage. The study was divided into three steps such as isolation of mRNA, synthesis of cDNA strand, and then real-time PCR to estimate the level of the gene expressed.

#### 4.5.2. Isolation of RNA and First-Strand cDNA Synthesis

Total mRNA was isolated by following the manufacturer’s protocol. In brief, brain tissue was properly homogenized in TRIzol^®®^ reagent, mixed with chloroform, and centrifuged at 13500 rpm (revolutions per minute) for 15 min at 4 °C. The upper aqueous supernatant was transferred into new tubes and isopropanol was added, mixed, and were incubated for 10 min at room temperature and later centrifuged for 10 min at 13,500 rpm at 4 °C. The supernatant was discarded and the pellets were rinsed with 75% ethanol. Then the pellets were left for air drying for 5 to 8 min. Finally, nuclease-free water was added to each tube to dissolve the mRNA pellet. The concentration and purity of the isolated mRNA were measured by using NanoDrop Spectrophotometer. The mRNA samples were converted into cDNA using Omniscript Reverse-transcription Kit (QIAGEN) according to the manufacturer’s protocol.

#### 4.5.3. StepOne^®®^ Real-time PCR

Gene expression study for the genes of our interest was measured by real-time quantitative RT-PCR (Step one Applied Biosystems) using QuantiTect SYRB Green dye (Qiagen, Valencia, CA).

All the primer sets were provided by Qiagen (npy: Dr_npy_1_SG QuantiTect Primer Assay (QT02205763).

HMGB1: Dr_hmgb1b_2_SG QuantiTect Primer Assay (Cat no: QT02088555)

TLR4: Dr_tlr4ba_va. 1_SG QuantiTect Primer Assay (Cat no: QT02198539)

NF-κB: Dr_nfkb1_2_SG QuantiTect Primer Assay (Cat no: QT02498762)

TNF-α: Dr_tnf_1_SG QuantiTect Primer Assay (Cat no: QT02097655)

IL-1: Dr_il1rapl1a_1_SG QuantiTect Primer Assay (Cat no: QT02131850)

BDNF: Dr_bdnf_1_SG QuantiTect Primer Assay (Cat no.QT02125326)

CREB_1: Dr_CREB_1 bpa_1_SG QuantiTect Primer Assay (Cat no. QT02197503)

NPY: Dr_npy_1_SG QuantiTect Primer Assay (Cat no: QT02205763)

eef1a1b: Dr_eef1a1b_2_SG QuantiTect Primer Assay (Cat no: QT02042684)

The PCR mixture contained 1X SYBR green PCR master mix (Qiagen), 0.7 µM each forward and reverse primers, and 1 µL of sample cDNA. Samples were incubated at 95 °C for 2 min before thermal cycling (40 cycles of 95 °C for 5 s and 60 °C for 15 s). Relative expression values of the above genes were obtained by normalizing threshold cycle (Ct) values of genes of interest against Ct value of eef1a1b (housekeeping gene) (2^^[Ct eef1a1b-Ct Gene of interest]^).

### 4.6. Brain Neurotransmitter Analysis

The level of the brain neurotransmitters such as GABA, Glutamate (Glu), and Acetylcholine (Ach) were analyzed using LC-MS/MS following the earlier documented study [21,51]. Stock solutions of the neurotransmitters were prepared in methanol (0.1% formic acid) to make up a final concentration of 1 mg/mL. The stock solutions were kept at 4 °C until needed. A range of 2000 to 125 parts per billion (ppb) was used for calibration. Each zebrafish’s brain was first homogenized in 200 µL of ice-cold methanol (1% formic acid). The homogenate was then vortex-mixed for 1 min and later centrifuged at 18,000× *g* for 10 min at 4 °C. Finally, the supernatant was pipetted into vials for LC-MS/MS analysis.

### 4.7. Zebrafish Brain Protein Estimation

Harvested zebrafish brains were lysed and homogenized with 200 ul of RIPA buffer (Thermo Fisher Scientific, USA) containing protease and phosphatase inhibitors (Thermo Fisher Scientific, USA). The homogenized solution was centrifuged at 500× *g* for 5 min at 4 °C. The supernatant was collected into a Protein Lo-Bind (Eppendorf) tube and the protein concentration was quantified using BCA Protein Assay Kit (Thermo Scientific Pierce) following the manufacturer’s instruction. Four zebrafish brain from each group has been pulled and the date set is generated as duplicate.

#### 4.7.1. In-Solution Tryptic Digestion

Proteins were denatured by using 25 µL of 100 mM ammonium bicarbonate (ABC), 25 µL of trifluoroethanol (TFE), and 1 µL of 200 mM 1,4-dithiothreitol (DTT). The samples were vortexed and incubated at 60 °C for 1 h. Next, proteins were alkylated by the addition of 4 µL 200 mM iodoacetamide (IAM) for 1 h in the dark at room temperature. Subsequently, 1 µL of 200 mM DTT was added to quench excess IAM and incubated for 1 h in the dark at room temperature. Next, 300 µL of water and 100 µL of ABC were added to dilute the denaturant and adjust the pH. Ten microliter of MS grade trypsin (20 µg/mL) was added and incubated for 14 h at 37 °C to digest the proteins. Finally, 1 µL of formic acid was added to terminate the reaction. The samples were dried in vacuum evaporator for overnight.

#### 4.7.2. Sample Desalting/Cleanup Using Spin Columns

Prior to LCMS/MS analysis, samples were cleaned-up/desalted using Pierce C18 Spin Column (Thermo Scientific, USA) following the manufacturer’s instruction. Desalted samples were re-concentrated in a vacuum concentrator and stored in −20 °C.

#### 4.7.3. Nanoflow-Ultra High-Performance Liquid Chromatography-Tandem Mass Spectrometry (LC-MS/MS)

Digested peptides were dissolved in 30 µL of 0.1% formic acid and centrifuged at 14000 rpm for 10 min. One microliter of the peptides was loaded into an Agilent C18, 300 Å large capacity chip (Agilent Technologies, Santa Clara, USA) mounted on an Agilent 1200 HPLC-Chip/MS interface, coupled with Agilent 6500 iFunnel quadrupole-time of flight (Q-TOF) LC/MS system. The chip was run at flow rate of 4 µL/min from Agilent 1200 Series Capillary pump and 0.5 µL/min from Agilent 1200 Series Nano Pump with 0.1% formic acid in water (solution A) and 90% acetonitrile in water with 0.1% formic acid (solution B). The peptides were eluted with multi-step gradients of 5–75% solution B: 5–75% solution B for 30 min, 75% solution for 9 min, and 75–5% solution B for 8 min. The ion polarity of the Q-TOF was set at positive, capillary voltage at 2050 V, fragmentor voltage at 360 V, gas temperature at 325 °C, and drying gas flow rate at 5 L/min. The spectra were acquired in auto MS/MS mode with a MS scan range of 110–3000 *m*/*z* and MS/MS scan range of 50–3000 *m*/*z*. Precursor charge state selection and preference was set as doubly, triply, or more than triply charged state, with the exclusion of precursors 299.294457 *m*/*z* (Z = 1) and 1221.990637 *m*/*z* (*Z* = 1) (reference ions).

#### 4.7.4. Protein Identification and Differential Expression Using PEAKS Bioinformatics Software

Protein identification and differential expression (label free quantification; LFQ) was performed with PEAKS studio 7.5 (Bioinformatics Solution Inc., Waterloo, Canada). UniProt Zebrafish (Danio rerio) (Dec 2018) database was used for protein identification and homology search. Carbamidomethylation was set as fixed modification with maximum missed cleavages at 3. Parent mass and fragment mass error tolerance were both set at 0.1 Da with monoisotopic as the precursor mass search type. Trypsin was selected as the digestion enzyme. Data filtering parameters were set at 1% false discover rate (FDR) and unique peptides ≥2. The LFQ parameters used were: mass error tolerance of 20 ppm, retention time shift tolerance of 6 min, and FDR threshold of 1%. Differentially expressed proteins between normal and epileptic zebrafish brain protein extracts were analyzed by hierarchical clustering. Heat map were generated by setting the protein significance ≥ 20 (which is equivalent to a *P*-value of 0.01), fold change ≥ 1, and has at least two unique peptides. ANOVA was set as the method for significance calculation. Experimental bias was taken into account by automatic normalization of protein ratios based on the total ion chromatogram (TIC).

### 4.8. Software and Instrumentation

Zebrafish swimming pattern was tracked by the Smart V3.0.05 tracking software (Pan Lab, Harvard apparatus). The Applied Biosystems StepOnePlus^TM^ Real-Time PCR System was used for the gene expression study.

### 4.9. Statistical Analysis:

For statistical analyses, Graph Pad Prism 8 software (Graph Pad Software, Inc.) was used. The locomotor behavior activity was analyzed as described above in zebrafish epilepsy behavior. Data are presented as means and standard errors of the mean (SEM). The results acquired were analyzed by T-test, one-way ANOVA, and subsequent Sidak’s multiple comparison test in order to assess the differences in seizure score, swimming pattern, gene expression levels, and neurotransmitters levels between all the groups. For all analyses, differences between a treatment group and the equivalent negative-control groups were considered statistically significant if the *p*-value was below 0.05 (*p* < 0.05).

## Figures and Tables

**Table 1:** Proposed seizure scoring for pilocarpine-induced seizure.

Score 0	Normal Swimming
Score 1	Jittery movement at the top of the tank
Score 2	Ataxia/Hyperactivity
Score 3	Circular movement, Circling around small area
Score 4	Erratic burst movement with loss of posture/Corkscrew swimming

Seizure score has been quantified based on the pilocarpine induced-seizure profile.

**Table 2:** List of 13 proteins identified by LCMS/MS from Zebrafish epileptic brain protein extract.

S.N.	Accession	−10lg*P*	Coverage (%)	#Peptides	#Unique	Avg. Mass	Protein Description	Biological Process (GO) from Uniprot and Protein Analysis Through Evolutionary Relationships (PANTHER)
1	P17561|EPD_DANRE	89.06	15	2	2	24,472	Ependymin	cell-matrix adhesion (GO:0007160)
2	tr|Q6DHB6|Q6DHB6_DANRE	71.15	6	4	3	49,994	Keratin 91	NA
3	tr|A0A2R8Q6V3|A0A2R8Q6V3_DANRE	38.54	4	2	2	57,453	Keratin type II cytoskeletal 8	NA
4	tr|A8WGN0|A8WGN0_DANRE	38.54	4	2	2	57,660	Keratin 8	NA
5	tr|Q6P3K5|Q6P3K5_DANRE	44.56	4	2	2	57,828	Krt5 protein	NA
6	tr|Q9PUB5|Q9PUB5_DANRE	44.56	4	2	2	58,587	Type II cytokeratin	fin regeneration (GO:0031101)
7	tr|F1R5A5|F1R5A5_DANRE	44.56	4	2	2	58,715	Keratin 5	
8	tr|A9JRN9|A9JRN9_DANRE	61.41	3	2	2	95,635	LOC794362 protein	axon development (GO:0061564)
9	tr|F1QCR7|F1QCR7_DANRE	61.41	3	2	2	95,577	Neurofilament medium polypeptide a	
10	tr|Q32PU7|Q32PU7_DANRE	83.35	8	3	1	49,717	Tubulin beta chain	microtubule-based process (GO:0007017)
11	tr|B8A516|B8A516_DANRE	68.72	6	2	2	49,969	Tubulin alpha chain	microtubule-based process (GO:0007017), microtubule cytoskeleton organization (GO:0000226), mitotic cell cycle (GO:0000278)
12	tr|Q502J6|Q502J6_DANRE	79.92	25	2	2	11,412	Gamma1a-synuclein	dopaminergic neuron differentiation (GO:0071542), larval locomotory behavior (GO:0008345)
13	tr|A0A2R8RP85|A0A2R8RP85_DANRE	79.92	25	2	2	11,442	Synuclein gamma b (breast cancer-specific protein 1)	

**Table 3:** List of 77 proteins identified by LCMS/MS analysis from normal zebrafish (control) group brain protein extract.

S.N.	Accession	−10lg*P*	Coverage (%)	#Peptides	#Unique	Avg. Mass	Protein Description	Biological Process (GO) From Uniprot and Protein Analysis Through Evolutionary Relationships (PANTHER)
1	tr|Q6P5M9|Q6P5M9_DANRE	153.04	50	16	2	49,787	Tubulin beta chain	Cellular comp. organization or biogenesis (GO:0071840)
2	Q90486|HBB1_DANRE	123.66	64	7	7	16,389	Hemoglobin subunit beta-1	Cellular comp. organization or biogenesis (GO:0071840), localization (GO: 0051179), Metabolic process (GO:0008152), response to stimulus (GO:0050896)
3	tr|Q6ZM12|Q6ZM12_DANRE	40.06	16	2	2	16,295	Hemoglobin beta adult 2	
4	tr|Q6ZM13|Q6ZM13_DANRE	80.62	29	3	2	15,413	Hemoglobin alpha adult 2	
5	tr|B3DG37|B3DG37_DANRE	123.66	64	7	7	16,389	Ba1 protein	
6	tr|Q803Z5|Q803Z5_DANRE	83.91	43	5	1	15,508	Hbaa1 protein	
7	tr|Q6DGK4|Q6DGK4_DANRE	40.06	16	2	2	16,279	Zgc:92880 protein	
8	tr|Q9DEU2|Q9DEU2_DANRE	112.71	10	8	2	112,703	Sodium/potassium-transporting ATPase subunit alpha	Biological regulation (GO:0065007), localization (GO: 0051179),
9	Q7ZVF9|ACTB2_DANRE	105.86	28	6	6	41,753	Actin cytoplasmic 2	Cellular process (GO:0009987), localization (GO: 0051179)
10	Q7ZVI7|ACTB1_DANRE	116.98	27	7	1	41,767	Actin cytoplasmic 1	
11	tr|A8WG05|A8WG05_DANRE	105.86	28	6	6	41,753	Bactin2 protein	
12	tr|B2GS08|B2GS08_DANRE	116.98	27	7	1	41,710	Bactin1 protein	
13	tr|R4GE02|R4GE02_DANRE	96.97	23	4	3	27,149	Si:ch211-113a14.11	Cellular comp. organization or biogenesis (GO:0071840)
14	tr|Q7ZU04|Q7ZU04_DANRE	90.53	20	5	5	42,916	Creatine kinase brain b	NA
15	tr|Q9I8N9|Q9I8N9_DANRE	81.04	29	3	3	14,918	Brain-type fatty acid-binding protein	NA
16	tr|A0A2R8Q1X2|A0A2R8Q1X2_DANRE	78.78	12	3	3	47,246	Enolase 1a (alpha)	Glycolytic process (GO:0006096)
17	tr|Q08BA1|Q08BA1_DANRE	77.71	7	3	3	59,744	ATP synthase subunit alpha	Metabolic process (GO:0008152), response to stimulus (GO:0050896)
18	tr|A8WGC6|A8WGC6_DANRE	53.73	6	2	2	55,130	ATP synthase subunit beta	
19	tr|Q6PC77|Q6PC77_DANRE	61.34	29	3	3	18,258	ATP synthase subunit d mitochondrial	
20	Q5MJ86|G3P2_DANRE	76.85	8	2	2	36,107	Glyceraldehyde-3-phosphate dehydrogenase 2	Metabolic process (GO:0008152)
21	tr|Q8JHI0|Q8JHI0_DANRE	67.61	12	3	3	32,763	Solute carrier family 25 (mitochondrial carrier; adenine nucleotide translocator) member 5	Transporter activity (GO:0005215)
22	tr|F1R5A5|F1R5A5_DANRE	67.55	5	4	2	58,715	Keratin 5	Fin regeneration (GO:0031101)
23	tr|I3IRY2|I3IRY2_DANRE	46.87	10	2	2	15,746	Keratin type 1 c19e (Fragment)	NA
24	tr|F1R8U0|F1R8U0_DANRE	46.87	4	2	2	47,785	Keratin 94	NA
25	tr|Q1RLR3|Q1RLR3_DANRE	46.87	3	2	2	50,931	Keratin 93	NA
26	tr|Q1LXJ9|Q1LXJ9_DANRE	46.87	3	2	2	49,934	Keratin type 1 c19e	NA
27	tr|Q24JW4|Q24JW4_DANRE	66.4	14	3	3	35,677	Flj13639	NA
28	Q90XG0|TPISB_DANRE	65.52	11	2	2	26,828	Triosephosphate isomerase B	Metabolic process (GO:0008152)
29	P17561|EPD_DANRE	55.6	12	2	2	24,472	Ependymin	Cell-matrix adhesion (GO:0007160)
30	Q7T356|143BB_DANRE	44.88	10	2	2	27,393	14-3-3 protein beta/alpha-B	Cell cycle (GO:0007049), Signal transduction (GO:0007165)
31	tr|A4FVM3|A4FVM3_DANRE	44.86	7	2	2	36,022	LOC557717 protein (Fragment)	Exocytosis (GO:0006887), Intracellular protein transport (GO:0006886), lysosomal transport (GO:0007041)
32	tr|A9JRN9|A9JRN9_DANRE	92.47	8	5	4	95,635	LOC794362 protein	ND
33	tr|A0A2R8QP59|A0A2R8QP59_DANRE	44.86	4	2	2	66,189	Syntaxin binding protein 1b	Localization (GO: 0051179), multicellular organismal process (GO: 0032501), signaling (GO:0023052).
34	tr|F1QM13|F1QM13_DANRE	44.86	4	2	2	67,075	Syntaxin-binding protein 1a	
35	tr|A2BGE0|A2BGE0_DANRE	44.86	3	2	2	68,575	Si:rp71-10d23.3	Localization (GO:0051179), multicellular organismal process (GO:0032501),
36	tr|F1QYN7|F1QYN7_DANRE	33.39	9	2	2	21,828	Myelin protein zero	Cellular process (GO:0009987)
37	tr|F8W3W8|F8W3W8_DANRE	111.06	60	9	9	10,776	Myelin basic protein a	
38	tr|A0A2R8QC30|A0A2R8QC30_DANRE	33.39	9	2	2	22,667	Uncharacterized protein	NA
39	tr|A0A2R8Q6P3|A0A2R8Q6P3_DANRE	33.39	8	2	2	25,013	Uncharacterized protein	NA
40	tr|Q0D294|Q0D294_DANRE	111.32	42	5	5	11,351	Histone H4	Nucleosome assemble (GO:0006334)
41	tr|Q6GQM9|Q6GQM9_DANRE	98.45	15	4	2	46,841	Eno2 protein	Metabolic process (GO:0008152)
42	Q6PI52|CALM_DANRE	98.01	52	6	6	16,838	Calmodulin	Biological regulation (GO:0065007), Cellular component organization or biogenesis (GO:0071840), cellular process (GO:0009987)
43	tr|Q7SZP4|Q7SZP4_DANRE	94.54	15	6	5	64,751	Zgc:65851	Nervous system development (GO:0007399)
44	tr|F1QCR7|F1QCR7_DANRE	92.47	8	5	4	95,577	Neurofilament medium polypeptide a	Axon development (GO:0061564)
45	tr|A0A2R8RRA6|A0A2R8RRA6_DANRE	92.13	8	4	4	74,958	Serotransferrin	Hemoglobin biosynthetic process (GO:0042541)
46	tr|F1Q8F1|F1Q8F1_DANRE	87.46	9	4	3	54,195	Internexin neuronal intermediate filament protein alpha b	Neuron projection morphogenesis (GO:0048812)
47	tr|Q58EH1|Q58EH1_DANRE	87.46	9	4	3	54,223	Gefiltin	Neuron projection morphogenesis (GO:0048812)
48	tr|Q5BJC7|Q5BJC7_DANRE	80.62	29	3	2	15,403	Si:xx-by187g17.5 protein	hydrogen peroxide catabolic process (GO:0042744), protein heterooligomerization (GO:0051291)
49	tr|A0A0B5JW41|A0A0B5JW41_DANRE	79.76	34	3	3	10,145	Cd59 (Fragment)	defense response to Gram-positive bacterium (GO:0050830)
50	tr|B3DFP9|B3DFP9_DANRE	78.8	50	4	4	15,537	Apolipoprotein A-II	chordate embryonic development (GO:0043009), nuclear division (GO:0000280)
51	tr|A0A0R4IKF0|A0A0R4IKF0_DANRE	65.95	18	4	4	30,140	Apolipoprotein A-Ib	biological regulation (GO:0065007), Cellular component organization or biogenesis (GO:0071840), localization (GO:0051179), metabolic process (GO:0008152), multicellular organismal process (GO:0032501)
52	Q804W2|PRV7_DANRE	78.02	33	3	3	12,029	Parvalbumin-7	biological regulation (GO:0065007)
53	tr|Q6TH32|Q6TH32_DANRE	72.15	21	2	2	18,771	Cofilin 1	developmental process (GO:0032502), metabolic process (GO:0008152)
54	Q9PVK4|LDHBA_DANRE	71.3	11	3	3	36,247	L-lactate dehydrogenase B-A chain	metabolic process (GO:0008152)
55	tr|Q7T334|Q7T334_DANRE	69.4	10	2	2	35,420	Malate dehydrogenase	
56	tr|U3JAS0|U3JAS0_DANRE	68.21	25	3	3	15,001	Synaptosomal-associated protein	cellular protein-containing complex assembly (GO:0034622), synaptic vesicle exocytosis (GO:0016079), vesicle fusion to plasma membrane (GO:0099500)
57	tr|Q7SX92|Q7SX92_DANRE	63.05	21	2	2	13,341	Beta-synuclein	dopaminergic neuron differentiation (GO:0071542), larval locomotory behavior (GO:0008345)
58	tr|E7F9E8|E7F9E8_DANRE	61.67	7	3	2	44,183	Neurofilament light polypeptide a	axon development (GO:0061564)
59	tr|A0A0N4STS4|A0A0N4STS4_DANRE	60.07	11	2	2	26,174	Ubiquitin B	modification-dependent protein catabolic process (GO:0019941), protein ubiquitination (GO:0016567)
60	tr|B3DLH2|B3DLH2_DANRE	60.07	5	2	2	51,501	Zgc:172187 protein	
61	tr|A0JPF1|A0JPF1_DANRE	60.07	5	2	2	59,921	Zgc:153686	
62	tr|Q7SXA3|Q7SXA3_DANRE	60.07	16	2	2	17,999	Ribosomal protein S27a	metabolic process (GO:0008152)
63	tr|B8JKN6|B8JKN6_DANRE	59.82	12	2	2	17,404	Peptidyl-prolyl cis-trans isomerase	protein refolding (GO:0042026)
64	tr|A0A2R8Q9S7|A0A2R8Q9S7_DANRE	58.58	60	2	2	6468	Si:dkey-46i9.1	NA
65	tr|Q0ZBR7|Q0ZBR7_DANRE	58.26	31	3	3	12,412	Macrophage migration inhibitory factor	auditory receptor cell development (GO:0060117), cell proliferation (GO:0008283), embryonic morphogenesis (GO:0048598), inner ear development (GO:0048839), negative regulation of apoptotic process (GO:0043066)
66	tr|Q502C8|Q502C8_DANRE	53.39	26	3	3	17,109	Peroxiredoxin 5	cell redox homeostasis (GO:0045454), cellular response to oxidative stress (GO:0034599), fin regeneration (GO:0031101), hydrogen peroxide catabolic process (GO:0042744)
67	tr|A0A0R4IG45|A0A0R4IG45_DANRE	52.38	2	2	2	94,588	Dynamin 1a	biological regulation (GO:0065007), cellular component organization or biogenesis (GO:0071840), cellular process (GO:0009987), localization (GO:0051179), metabolic process (GO:0008152)
68	tr|E9QF63|E9QF63_DANRE	52.38	2	2	2	95,446	Dynamin 1b	
69	tr|Q6TNV0|Q6TNV0_DANRE	51.28	11	2	2	19,385	Cytochrome c oxidase subunit 4I1	metabolic process (GO:0008152)
70	tr|Q4VBT9|Q4VBT9_DANRE	51.28	11	2	2	19,443	Cox4i1 protein	metabolic process (GO:0008152), mitochondrial electron transport, cytochrome c to oxygen (GO:0006123)
71	tr|Q5TZ35|Q5TZ35_DANRE	50.73	13	2	2	22,261	Visinin-like 1b	NA
72	tr|A0A2R8RID5|A0A2R8RID5_DANRE	50.34	5	2	2	84,805	Aconitate hydratase mitochondrial	tricarboxylic acid cycle (GO:0006099)
	Q6IQM2|CYC_DANRE	49.91	18	2	2	11,456	Cytochrome c	mitochondrial electron transport, cytochrome c to oxygen (GO:0006123)
73	Q8JH70|ALDCB_DANRE	48.6	9	2	2	39,259	Fructose-bisphosphate aldolase C-B	glycolytic process (GO:0006096)
74	tr|Q05AL9|Q05AL9_DANRE	46.87	4	2	2	47,758	Zgc:153629 protein	NA
75	tr|Q1JQ08|Q1JQ08_DANRE	46.87	3	2	2	51,584	Si:dkeyp-113d7.4 protein (Fragment)	NA
76	tr|F1R9V3|F1R9V3_DANRE	42.3	4	2	2	71,094	Si:dkey-4p15.3	cellular process (GO:0009987), localization (GO:0051179), response to stimulus (GO:0050896)
77	tr|A0A0R4IMF8|A0A0R4IMF8_DANRE	42.3	4	2	2	70,145	Heat shock cognate 71 kDa protein	cellular process (GO:0009987), localization (GO:0051179), response to stimulus (GO:0050896)

**Table 4:** Proteins identified from zebrafish brain extract (control vs epileptic) with differential expression based on label free quantification approach. Biological process for the proteins were identified using PANTHER-GO classification system software.

S.N.	Accession	Group Profile (Ratio, Control: Epileptic)	Description	PANTHER GO-Slim Biological Process (*Danio rerio*)
1	tr|F8W3W8|F8W3W8_DANRE	1.00:0.09	Myelin basic protein a	No Match
2	tr|Q8AY63|Q8AY63_DANRE	1.00:0	Brain-subtype creatine kinase	No Match
3	tr|F1R3D3|F1R3D3_DANRE	1.00:0.02	Glyceraldehyde-3-phosphate dehydrogenase	No Match
4	tr|R4GE02|R4GE02_DANRE	1.00:0.03	Si:ch211-113a14.11	Cellular component organization or biogenesis (GO:0071840
5	tr|Q9I8N9|Q9I8N9_DANRE	1.00:0.04	Brain-type fatty acid-binding protein	No Match
6	tr|Q6IQP5|Q6IQP5_DANRE	1.00:0	Enolase 1 (Alpha)	No Match
7	tr|Q1LWD7|Q1LWD7_DANRE	1.00:0.02	Parvalbumin	No Match
8	tr|A0A0R4IS04|A0A0R4IS04_DANRE	1.00:0	Myelin protein zero	No Match
9	tr|B3DG37|B3DG37_DANRE	1.00:0.03	Ba1 protein	No Match
10	tr|E9QJ96|E9QJ96_DANRE	1.00:0.03	14-3-3 protein beta/alpha-A (Fragment)	No Match
11	tr|Q08BA1|Q08BA1_DANRE	1.00:0.08	ATP synthase subunit alpha	Metabolic process (GO:0008152), Response to stimulus (GO:0050896)
12	tr|Q6PC77|Q6PC77_DANRE	1.00:0	ATP synthase subunit d mitochondrial	Metabolic process (GO:0008152)
13	tr|A8WGC6|A8WGC6_DANRE	1.00:0.04	ATP synthase subunit	Metabolic process (GO:0008152)
14	tr|Q24JW4|Q24JW4_DANRE	1.00:0	Flj13639	Multicellular organismal process (GO:0032501)
15	tr|A3KPR4|A3KPR4_DANRE	1.00:0.00	Histone H4	No Match
16	tr|Q7T334|Q7T334_DANRE	1.00:0	Malate dehydrogenase	Metabolic process (GO:0008152)
17	tr|Q6ZM12|Q6ZM12_DANRE	1.00:0	Hemoglobin beta adult 2	localization (GO:0051179), Metabolic process (GO:0008152), Response to stimulus (GO:0050896)
18	tr|A0A0B5JW41|A0A0B5JW41_DANRE	1.00:0.01	Cd59 (Fragment)	No Match
19	tr|A0A0R4IKF0|A0A0R4IKF0_DANRE	1.00:0	Apolipoprotein A-Ib	Cellular component organization or biogenesis (GO:0071840), Biological regulation (GO:0065007), localization (GO:0051179), Metabolic process (GO:0008152), Multicellular organismal process (GO:0032501)

## References

[1] Fisher R.S., Acevedo C., Arzimanoglou A., Bogacz A., Cross J.H., Elger C.E., Engel J., Forsgren L., French J.A., Glynn M. (2014). ILAE official report: A practical clinical definition of epilepsy. Epilepsia.

[2] Vezzani A., Aronica E., Mazarati A., Pittman Q.J. (2013). Epilepsy and brain inflammation. Exp. Neurol..

[3] Paudel Y.N., Shaikh M.F., Shah S., Kumari Y., Othman I. (2018). Role of inflammation in epilepsy and neurobehavioral comorbidities: Implication for therapy. Eur. J. Pharmacol..

[4] Van Vliet E.A., Aronica E., Vezzani A., Ravizza T. (2018). Neuroinflammatory pathways as treatment targets and biomarker candidates in epilepsy: Emerging evidence from preclinical and clinical studies. Neuropathol. Appl. Neurobiol..

[5] Ravizza T., Terrone G., Salamone A., Frigerio F., Balosso S., Antoine D.J., Vezzani A. (2017). High mobility group box 1 is a novel pathogenic factor and a mechanistic biomarker for epilepsy. Brain Behav. Immun..

[6] Paudel Y.N., Shaikh M., Serrano Á.A., Kumari Y., Aleksovska K., Alvim M.K.M., Chakraborti A., Othman I.B. (2018). HMGB1: A Common Biomarker and Potential Target for TBI, Neuroinflammation, Epilepsy and Cognitive Dysfunction. Front. Neurosci..

[7] Paudel Y.N., Angelopoulou E., Piperi C., Balasubramaniam V.R., Othman I., Shaikh M.F. (2019). Enlightening the role of high mobility group box 1 (HMGB1) in inflammation: Updates on receptor signalling. Eur. J. Pharmacol..

[8] Shams S., Rihel J., Ortiz J.G., Gerlai R. (2017). The zebrafish as a promising tool for modeling human brain disorders: A review based upon an IBNS Symposium. Neurosci. Biobehav. Rev..

[9] Copmans D.l., Rateb M., Tabudravu J.N., Pérez-Bonilla M., Dirkx N., Vallorani R., Diaz C., Pérez del Palacio J., Smith A.J., Ebel R. (2018). Zebrafish-Based Discovery of Antiseizure Compounds from the Red Sea: Pseurotin A2 and Azaspirofuran A. ACS Chem. Neurosci..

[10] Norton W., Bally-Cuif L. (2010). Adult zebrafish as a model organism for behavioural genetics. BMC Neurosci..

[11] Mussulini B.H.M., Leite C.E., Zenki K.C., Moro L., Baggio S., Rico E.P., Rosemberg D.B., Dias R.D., Souza T.M., Calcagnotto M.E. (2013). Seizures induced by pentylenetetrazole in the adult zebrafish: A detailed behavioral characterization. PLoS ONE.

[12] Gross A., Benninger F., Madar R., Illouz T., Griffioen K., Steiner I., Offen D., Okun E. (2017). Toll-like receptor 3 deficiency decreases epileptogenesis in a pilocarpine model of SE-induced epilepsy in mice. Epilepsia.

[13] Lin W., Huang W., Chen S., Lin M., Huang Q., Huang H. (2017). The Role of 5-HTR6 in Mossy Fiber Sprouting: Activating Fyn and p-ERK1/2 in Pilocarpine-Induced Chronic Epileptic Rats. Cell. Physiol. Biochem..

[14] Lee M., Young Choi B., Won Suh S. (2018). Unexpected effects of acetylcholine precursors on pilocarpine seizure-induced neuronal death. Curr. Neuropharmacol..

[15] Fu L., Liu K., Wake H., Teshigawara K., Yoshino T., Takahashi H., Mori S., Nishibori M. (2017). Therapeutic effects of anti-HMGB1 monoclonal antibody on pilocarpine-induced status epilepticus in mice. Sci. Rep..

[16] Mussulini B.H.M., Vizuete A.F.K., Braga M., Moro L., Baggio S., Santos E., Lazzarotto G., Zenki K.C., Pettenuzzo L., da Rocha J.B.T. (2018). Forebrain glutamate uptake and behavioral parameters are altered in adult zebrafish after the induction of Status Epilepticus by kainic acid. Neurotoxicology.

[17] Vermoesen K., Serruys A.-S.K., Loyens E., Afrikanova T., Massie A., Schallier A., Michotte Y., Crawford A.D., Esguerra C.V., de Witte P.A. (2011). Assessment of the convulsant liability of antidepressants using zebrafish and mouse seizure models. Epilepsy Behav..

[18] Winter M.J., Windell D., Metz J., Matthews P., Pinion J., Brown J.T., Hetheridge M.J., Ball J.S., Owen S.F., Redfern W.S. (2017). 4-dimensional functional profiling in the convulsant-treated larval zebrafish brain. Sci. Rep..

[19] Kalueff A.V., Gebhardt M., Stewart A.M., Cachat J.M., Brimmer M., Chawla J.S., Craddock C., Kyzar E.J., Roth A., Landsman S. (2013). Towards a comprehensive catalog of zebrafish behavior 1.0 and beyond. Zebrafish.

[20] Baraban S., Taylor M., Castro P., Baier H. (2005). Pentylenetetrazole induced changes in zebrafish behavior, neural activity and c-fos expression. Neuroscience.

[21] Kundap U.P., Kumari Y., Othman I., Shaikh M. (2017). Zebrafish as a model for epilepsy-induced cognitive dysfunction: A pharmacological, biochemical and behavioral approach. Front. Pharmacol..

[22] Mezzomo N.J., Fontana B.D., Kalueff A.V., Barcellos L.J., Rosemberg D.B. (2018). Understanding taurine CNS activity using alternative zebrafish models. Neurosci. Biobehav. Rev..

[23] Choo B.K.M., Kundap U.P., Kumari Y., Hue S.-M., Othman I., Shaikh M.F. (2018). Orthosiphon stamineus leaf extract affects TNF-α and seizures in a zebrafish model. Front. Pharmacol..

[24] Amini E., Golpich M., Farjam A.S., Kamalidehghan B., Mohamed Z., Ibrahim N.M., Ahmadiani A., Raymond A.A. (2018). Brain Lipopolysaccharide Preconditioning-Induced Gene Reprogramming Mediates a Tolerance State in Electroconvulsive Shock Model of Epilepsy. Front. Pharmacol..

[25] Vezzani A., Granata T. (2005). Brain inflammation in epilepsy: Experimental and clinical evidence. Epilepsia.

[26] Vezzani A., French J., Bartfai T., Baram T.Z. (2011). The role of inflammation in epilepsy. Nat. Rev. Neurol..

[27] Maroso M., Balosso S., Ravizza T., Liu J., Aronica E., Iyer A.M., Rossetti C., Molteni M., Casalgrandi M., Manfredi A.A. (2010). Toll-like receptor 4 and high-mobility group box-1 are involved in ictogenesis and can be targeted to reduce seizures. Nat. Med..

[28] Ying C., Ying L., Yanxia L., Le W., Lili C. (2019). High mobility group box 1 antibody represses autophagy and alleviates hippocampus damage in pilocarpine-induced mouse epilepsy model. Acta Histochem..

[29] Shi Y., Zhang L., Teng J., Miao W. (2018). HMGB1 mediates microglia activation via the TLR4/NF-κB pathway in coriaria lactone induced epilepsy. Mol. Med. Rep..

[30] Liu A.-H., Wu Y.-T., Wang Y.-P. (2017). MicroRNA-129-5p inhibits the development of autoimmune encephalomyelitis-related epilepsy by targeting HMGB1 through the TLR4/NF-kB signaling pathway. Brain Res. Bull..

[31] Qu Z., Jia L., Xie T., Zhen J., Si P., Cui Z., Xue Y., Sun C., Wang W. (2019). (−)-Epigallocatechin-3-Gallate Protects Against Lithium-Pilocarpine-Induced Epilepsy by Inhibiting the Toll-Like Receptor 4 (TLR4)/Nuclear Factor-κB (NF-κB) Signaling Pathway. Med. Sci. Mon..

[32] Ashhab M.U., Omran A., Kong H., Gan N., He F., Peng J., Yin F. (2013). Expressions of tumor necrosis factor alpha and microRNA-155 in immature rat model of status epilepticus and children with mesial temporal lobe epilepsy. J. Mol. Neurosci..

[33] Rijkers K., Majoie H., Hoogland G., Kenis G., De Baets M., Vles J. (2009). The role of interleukin-1 in seizures and epilepsy: A critical review. Exp. Neurol..

[34] De Simoni M.G., Perego C., Ravizza T., Moneta D., Conti M., Marchesi F., De Luigi A., Garattini S., Vezzani A. (2000). Inflammatory cytokines and related genes are induced in the rat hippocampus by limbic status epilepticus. Eur. J. Neurosci..

[35] Gall C.M. (1993). Seizure-induced changes in neurotrophin expression: Implications for epilepsy. Exp. Neurol..

[36] Zhu X., Han X., Blendy J.A., Porter B.E. (2012). Decreased CREB levels suppress epilepsy. Neurobiol. Dis..

[37] Kandel E.R. (2012). The molecular biology of memory: cAMP, PKA, CRE, CREB-1, CREB-2, and CPEB. Mol. Brain.

[38] Noe F., Vaghi V., Balducci C., Fitzsimons H., Bland R., Zardoni D., Sperk G., Carli M., During M., Vezzani A. (2010). Anticonvulsant effects and behavioural outcomes of rAAV serotype 1 vector-mediated neuropeptide Y overexpression in rat hippocampus. Gene Ther..

[39] Gøtzsche C., Woldbye D. (2016). The role of NPY in learning and memory. Neuropeptides.

[40] Luo J., Min S., Wei K., Li P., Dong J., Liu Y.-f. (2011). Propofol protects against impairment of learning-memory and imbalance of hippocampal Glu/GABA induced by electroconvulsive shock in depressed rats. J. Anesth..

[41] Kaila K., Ruusuvuori E., Seja P., Voipio J., Puskarjov M. (2014). GABA actions and ionic plasticity in epilepsy. Curr. Opin. Neurobiol..

[42] Choo B.K.M., Kundap U.P., bin Johan Arief M.F., Kumari Y., Yap J.L., Wong C.P., Othman I., Shaikh M.F. (2019). Effect of newer anti-epileptic drugs (AEDs) on the cognitive status in pentylenetetrazol induced seizures in a zebrafish model. Prog. Neuro-Psychopharmacol. Biol. Psychiatry.

[43] Miller H.P., Levey A.I., Rothstein J.D., Tzingounis A.V., Conn P.J. (1997). Alterations in glutamate transporter protein levels in kindling-induced epilepsy. J. Neurochem..

[44] Chapman A.G. (2000). Glutamate and epilepsy. J. Nutr..

[45] Holmes G.L. (2002). Seizure-induced neuronal injury: Animal data. Neurology.

[46] Izquierdo I., Medina J.H. (1997). Memory formation: The sequence of biochemical events in the hippocampus and its connection to activity in other brain structures. Neurobiol. Learn. Memory.

[47] Atzori M., Kanold P., Pineda J.C., FLORES-HERNANDEZ J. (2003). Dopamine-Acetylcholine Interactions in the Modulation of Glutamate Release. Ann. N. Y. Acad. Sci..

[48] Persike D., Marques-Carneiro J., Stein M., Yacubian E., Centeno R., Canzian M., Fernandes M. (2018). Altered Proteins in the Hippocampus of Patients with Mesial Temporal Lobe Epilepsy. Pharmaceuticals.

[49] Yang J., Czech T., Felizardo M., Baumgartner C., Lubec G. (2006). Aberrant expression of cytoskeleton proteins in hippocampus from patients with mesial temporal lobe epilepsy. Amino Acids.

[50] Yang Q., Wang S., Karlsson J.-E., Hamberger A., Haglid K.G. (1995). Phosphorylated and non-phosphorylated neurofilament proteins: Distribution in the rat hippocampus and early changes after kainic acid induced seizures. J. Chem. Neuroanat..

[51] Kundap U.P., Paudel Y.N., Kumari Y., Othman I., Shaikh M.F. (2019). Embelin prevents seizure and associated cognitive impairments in a pentylenetetrazole-induced kindling zebrafish model. Front. Pharmacol..

